# Effect of thermal softening of double-lumen endobronchial tubes on postoperative sore throat in patients with prior SARS-CoV-2 infection: a randomized controlled trial

**DOI:** 10.1186/s12871-023-02363-y

**Published:** 2023-12-07

**Authors:** Wenlong Yan, Jianyue Cai, Chenchen Zhu, Yu Chen, Jun Fang, Hui Xu, Xiaojing Zheng, Yingting Zhou, Yanhu Xie, Min Zhang, Shuhua Shu

**Affiliations:** Department of Anesthesiology, The First Affiliated Hospital of USTC, Division of Life Sciences and Medicine, University of Science and Technology of China, Hefei, Anhui, 230036 China

**Keywords:** One-lung ventilation, Postoperative sore throat, Thermalsoftening, DLT intubation, SARS-CoV-2 Infection

## Abstract

**Background:**

The incidence of postoperative sore throat (POST) after tracheal intubation using double-lumen endobronchial tubes (DLTs) is higher in patients with prior severe acute respiratory syndrome coronavirus 2 (SARS-CoV-2) infection than in the general population. This prospective trial was conducted to determine whether thermal softening of DLTs could decrease the incidence of POST or other airway injuries in patients with prior SARS-CoV-2 infection.

**Methods:**

A total of 120 patients with prior SARS-CoV-2 infection undergoing thoracoscopic surgery were randomly assigned to two groups (n = 60 each). In the thermal softening group, the distal portion of the DLT was placed in thermostatic saline (50 °C) for 10 min before endotracheal intubation. In the control group, the distal portion of the DLT was placed in room temperature saline for 10 min before endotracheal intubation. The incidence and severity of POST and hoarseness were assessed at 1, 6 and 24 h postoperatively. The primary outcomes were the incidence and severity of POST at 6 h postoperatively. The secondary outcomes were the incidence and severity of hoarseness, vocal cord and tracheal injuries, and hemodynamic changes in patients at intubation.

**Results:**

The incidence of POST at 6 h postoperatively was greater in the control group than in the thermal softening group [41 (68%) vs. 22 (37%), *P* = 0.001]. The overall incidence of POST at 24 h postoperatively was greater in the control group than in the thermal softening group [46 (76%) vs. 24 (40%), *P* < 0.001]. The overall incidence of tracheal injuries was also greater in the control group than in the thermal softening group (*P* = 0.016). Vocal cord injuries occurred more frequently in the control group than in the thermal softening group (*P* = 0.006).

**Conclusion:**

Thermal softening of DLTs before intubation can reduce the incidence of POST and airway injuries in patients with prior SARS-CoV-2 infection undergoing DLT insertion.

**Trial registration:**

This trial has been registered at www.chictr.org.cn (registration number: ChiCTR2200066821; registration date: December 19, 2022).

## Background

Most anesthesiologists prefer using the double-lumen endobronchial tube (DLT) over the single-lumen endobronchial tube (SLT) with bronchial blockers because the former can enable faster localization and lung deflation and can ventilate each lung individually [1]. However, tracheal intubation with DLT may cause a higher incidence of airway injuries and postoperative sore throat (POST) than that with SLT [1, 2]. This is probably because compared to SLTs, DLTs have a harder texture and thicker diameter and are inserted deeper into the airway [3, 4]. POST frequently occurs as a self-limiting complication; however, it can reduce patient satisfaction and delay postoperative recovery [5]. Therefore, preventive techniques are required to reduce potential airway injuries and complications related to intubation with DLTs.

Severe acute respiratory syndrome corona virus 2 (SARS-CoV-2) infection causes local mucosal epithelial damage in the pharynx and trachea, resulting in nonspecific inflammation; moreover, in some patients with poor immunity, SARS-CoV-2 infection can persist and lead to chronic inflammation [6]. SARS-CoV-2 primarily infects the airway and can persist in the airway lumen for a long time [7], which may lead to increased airway sensitivity and a higher incidence of intubation-related complications in patients [8].

Various nonpharmacological and pharmacological approaches have been used to decrease the incidence of POST [3, 4, 9, 10]. Nonpharmacological approaches include using small-sized endotracheal tubes (ETTs), monitoring the depth of the protective sleeve over the ET, engaging a skilled anesthesiologist to perform the intubation procedure, using a video laryngoscope, etc. [11]. Thermal softening of the DLT can reduce its stiffness and increase the flexibility of the tube; thus, this approach has been used to reduce the incidence of POST and airway injuries during intubation with DLTs [3]. However, the effect of thermal softening of DLT in patients with prior SARS-CoV-2 infection has not been adequately studied.

We hypothesized that thermal softening of DLTs before intubation may reduce the incidence of intubation-related airway complications in patients with prior SARS-CoV-2 infection undergoing one-lung anesthesia.

## Methods

### Study design

A randomized controlled trial was conducted in the First Affiliated Hospital of the University of Science and Technology of China (USTC). The study was approved by the ethics committee of the First Affiliated Hospital of USTC (approval no. KY2022-337). This trial was registered in the Chinese Registry of Clinical Trials (http://www.chictr.org.cn; registration no. ChiCTR2200066821) on 19/12/2022. Prior to the study, written informed consent was obtained from all participants.

We enrolled patients who were aged 18–75 years, had ASA physical status I-III, had SARS-CoV-2 infection 3 months prior to surgery, and had received elective thoracoscopic lung surgery using DLTs from December 2022 to April 2023. The following patients were excluded: (1) those who refused to participate; (2) those with a recent incidence of sore throat or upper respiratory tract infection orhoarseness; (3) those with a Mallampati score of 4; and (4) those with a history of difficult airway.

**Fig. 1:**
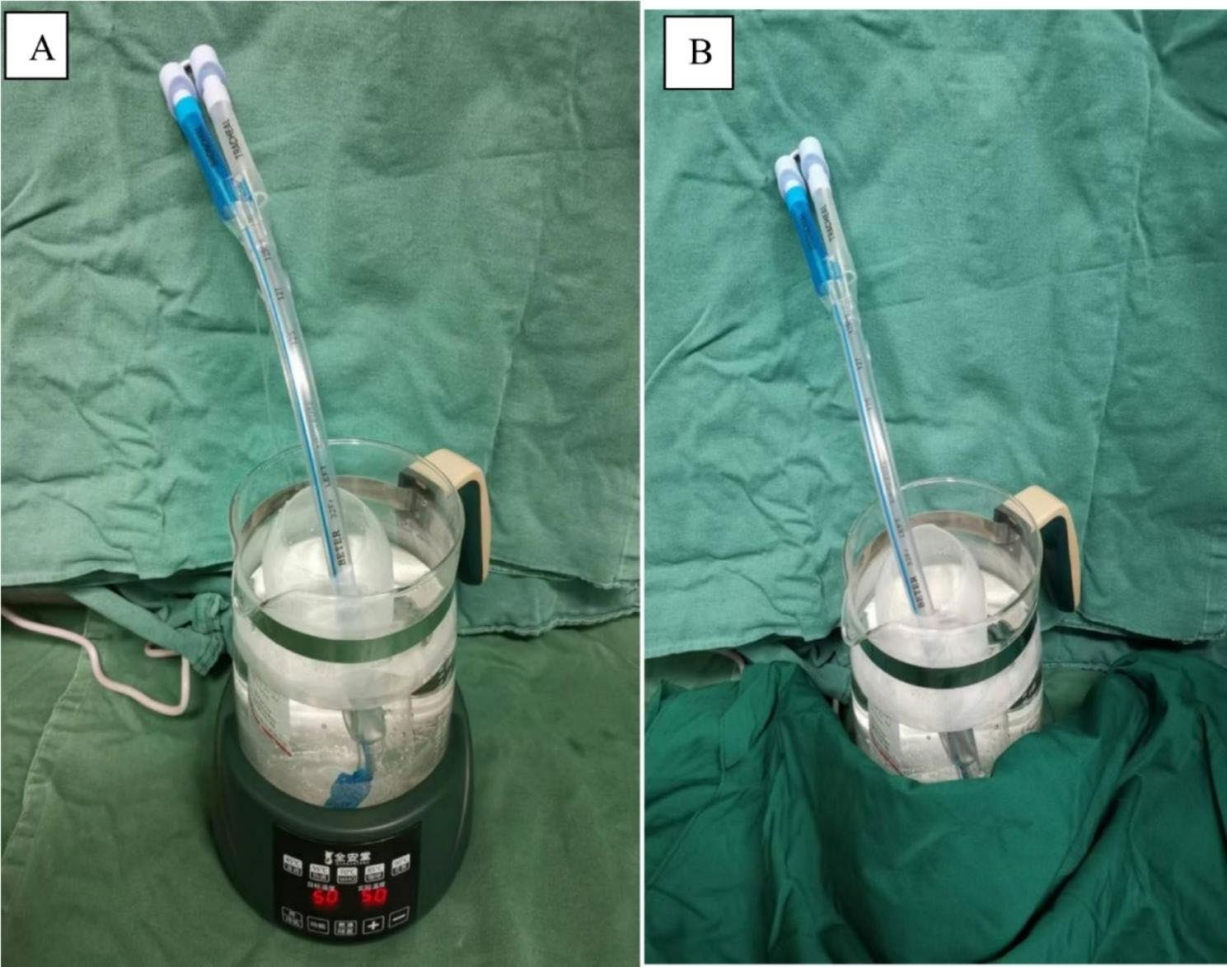
The thermal softening process for the DLT. **(A)** The distal portion of the DLT was placed in a sterile saline solution bottle, and the bottle was kept on a constant temperature kettle whose temperature could be adjusted. **(B)** The constant temperature kettle was covered with a layer of cloth to blind hospital staff regarding group allocation

Each individual was randomly assigned to the thermal softening group or the control group in a 1:1 ratio on the basis of a random table generated by a computer randomization program. The random sequence was placed in opaque, sealed envelopes. Patients, anesthesiologists, surgical team members, nurses, and postoperative follow-up evaluators were blinded to group assignment. After patients entered the operating room, the envelope was opened by a researcher who was also blinded to group allocation. The researcher prepared and pretreated all DLTs(Tuoren Best Medical Equipment Company, Henan, China). The size of the DLT depended on the patients’ gender and height [12]. The researcher first evacuated the air of the tracheal cuff and bronchial cuff and then immersed the distal part of the DLTs in a sterile saline solution for 10 min (Fig. 1). The temperature of the saline was maintained at 50 °C for the thermal softening group or at room temperature for the control group; a thermostatic kettle was used to measure saline temperature. The temperature was regulated at the desired level, and following the completion of thermal softening, the DLTs were removed from the saline solution bottle before tracheal intubation.

Once the patient entered the operating room, blood oxygen saturation, electrocardiography, heart rate, noninvasive blood pressure, and bispectral index (BIS) were continuously monitored. Invasive arterial blood pressure was monitored by radial artery puncture and catheterization after local anesthesia.

Anesthesia was induced using midazolam (0.05 mg/kg), etomidate (0.3 mg/kg), sufentanil (0.5 µg/kg), and cisatracurium (0.2 mg/kg). An otorhinolaryngologist blinded to group allocation evaluated the vocal cords of the patients before tracheal intubation by using a video laryngoscope and acquired photographs for subsequent comparison. Tracheal intubation was performed by an anesthesiologist with experience in performing over 500 DLT insertions by using a video laryngoscope (INSIDES Medical Technology Company, Shenzhen, China). Endotracheal intubation was performed with a disposable polyvinyl chloride DLT by using a video laryngoscope. After inserting the tip of the bronchus forward through the glottis, the anesthesiologist removed the tube core and continue pushing forward.

If substantial airway resistance was encountered, the anesthesiologist rotated the DLT counterclockwise or clockwise by 90° and then pushed it forward. If severe resistance was still experienced, the anesthesiologist again rotated the DLT counterclockwise or clockwise by 90° and subsequently advanced the DLT [4]. The anesthesiologist adjusted the position of the patient’s head and used a fiberoptic bronchoscope(FOB)(UE Medical Company, Zhejiang, China) to evaluate and adjust the position of the DLT [13]. After the patient was placed in the lateral position, the position of the DLT was again confirmed using the FOB. The pressure in the tracheal and bronchial cuff was adjusted to less than 25 cmH_2_O and 44cmH_2_O, respectively, by using a cuff pressure monitoring device [14]. General anesthesia was maintained using propofol at 4–8 mgkg^− 1^ h^− 1^ and remifentanil at 0.1–0.3 µgkg^− 1^ min^− 1^. The infusion rate of propofol and remifentanil was adjusted to control the depth of anesthesia within a BIS of 40 to 60.

Thirty minutes before the completion of the surgery, the patients were intravenously administered 1 mg kg^− 1^ flurbiprofen axetil and 0.1 mg kg^− 1^oxycodone for postoperative pain management. After surgery, all patients were shifted to the postanesthesia care unit(PACU). The patients were treated with 0.05 mg kg^− 1^ neostigmine and 0.01 mg kg^− 1^ atropine to antagonize residual neuromuscular block. Patient-controlled intravenous analgesia(PCIA) infusion was initiated after extubation for all patients. The PCIA infusion comprised 1 µg/mL sufentanil and 1 mg/mL flurbiprofen in normal saline at a continuous infusion rate of 2 mL h^− 1^ with a 2-mL bolus and a lockout interval of 15 min.

### Measurement of outcomes

The Mallampati grade was assessed preoperatively by ananesthesiologist blinded to the study details. The anesthesiologist used a video laryngoscope to acquire photographs and record the images of the vocal cords during endotracheal intubation; the laryngoscopy field of view was graded using the Cormack and Lehane scoring system. The percentage of glottis exposure was recorded in the range of 0–100%. The resistance encountered by the anesthesiologist during the insertion of DLTs was categorized into four levels: no resistance, mild resistance, moderate resistance, and severe resistance. The intubation time was defined as the time between the insertion of the laryngoscope into the patient’s mouth and the removal of the laryngoscope. The following hemodynamic parameters were measured: heart rate and mean arterial pressure before intubation and 2 min after intubation.

Trachea and vocal cord injuries related to DLT intubation were assessed by a blinded otorhinolaryngologist with more than 5 years of experience in using an FOB. Before removing the DLT, the otorhinolaryngologist inserted the FOB into the DLT and evaluated trachea and vocal cord injuries during extubation. Tracheal injury was assessed using the tracheal injury clinical scoring systembased on mucosal changes [15]. Vocal cord injury was assessed based on the following characteristics: (1) edema, swollen mucosa at the vocal folds; (2) erythema, redness of the mucosa with inflammatory swelling of the surrounding area; and (3) hematoma, bleeding of the vocal cord [16, 17].

POST was observed in the PACU at 1 h after the surgery and in the ward at 6 and 24 h after the surgery by a researcher blinded to group allocation. The severity of POST was assessed as follows: 0, no sore throat; 1, mild (less than that observed in common cold); 2, moderate (similar to that observed in common cold); and 3, severe (more than that observed in common cold) [18, 19]. The incidence and severity of hoarseness were also recorded. Hoarseness was assessed as follows: 0, no hoarseness; 1, mild (no hoarseness at the time of interview but was present previously); 2, moderate (perceived only by the patient); and 3, severe (recognizable at the time of interview) [18, 19].

### Statistical analysis

In the study of Parket al. [20], 58% of the patients in the control group complained of sore throat at 6 h following DLT insertion. A 50% reduction in the incidence of POST was considered clinically significant. Therefore, 52 patients were required in each group, with an alpha level of 0.05 and astatistical power of 80%. A 10% drop-out rate was allowed; thus, 60 patients were required in each group.

SPSS version 25.0 software (IBM, Chicago, IL, USA) was used for all statistical analysis. For continuous data, the Kolmogorov–Smirnov test was used to determine data normality. Normally distributed data were reported as mean ± SD and evaluated using an independent Student’s t-test for equal variance. Non-normally distributed interval and ordinal data were expressed as median (interquartile range) and evaluated using Mann–Whiney *U* test. Categorical variables were expressed as absolute numbers (%) and analyzed by the χ^2^ test. For all analyses, *P* < 0.05 (two-tailed) was considered significant.

## Results

**Fig. 2:**
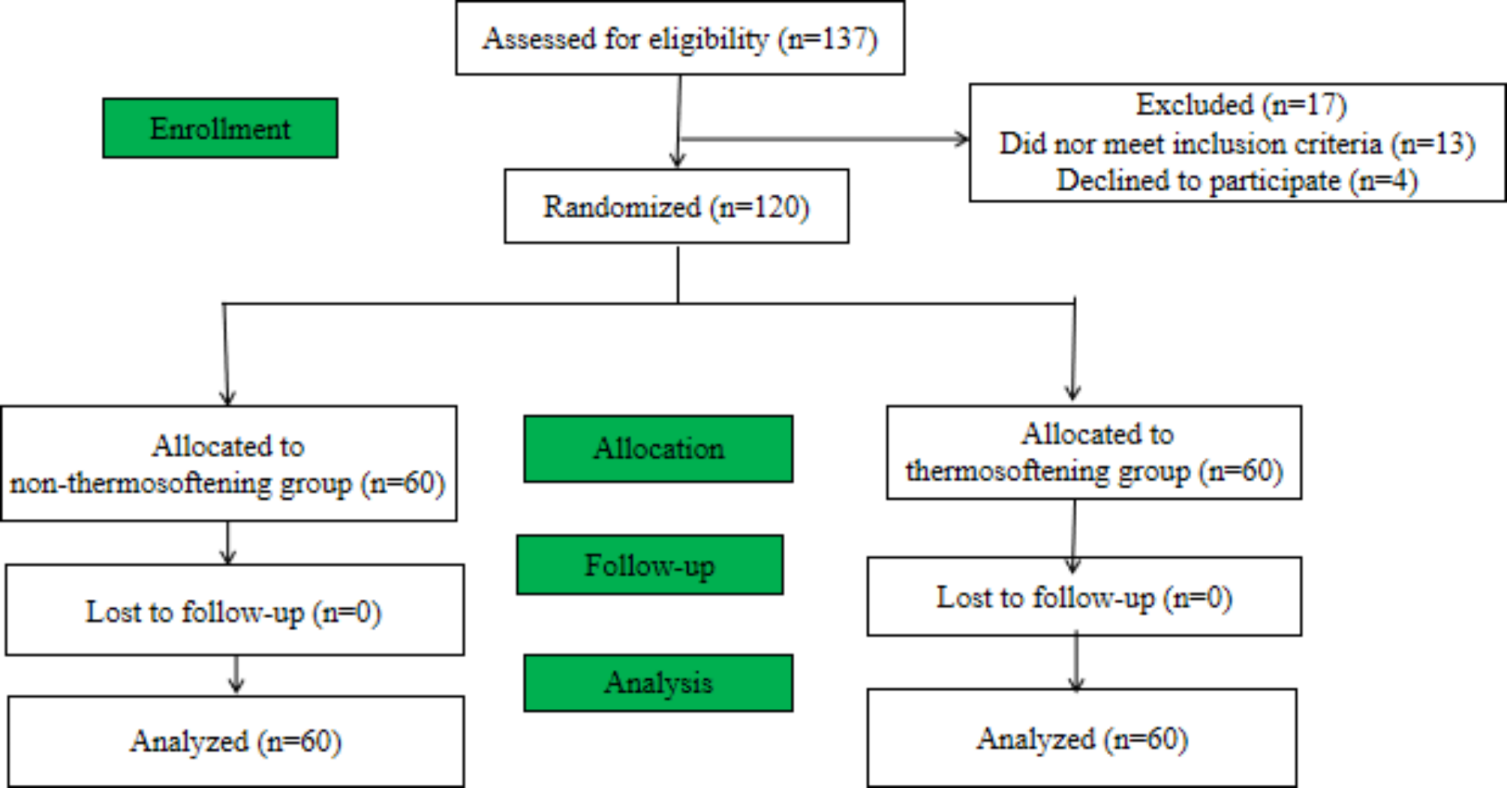
Flow diagram of the study design

The study recruited 137 patients who were treated between December 2022 and April 2023, and 17 patients were excluded (Fig. 2).

Both groups showed no significant differences in patient characteristics (Table 1).

**Table 1:** Comparison of general characteristics of the two groups ($\bar x \pm s,$  *n* = 60)

Baseline characteristics	Controlgroup (*n* = 60)	Thermal softening group(*n* = 60)	*P*
Age (years)	59 ± 10	60 ± 11	0.692
Sex (male/female)	31/29	33/27	0.568
Weight(kg)	60 ± 8	63 ± 10	0.837
Height(cm)	161 ± 8	163 ± 7	0.421
BMI (kg/m^2^)	23.1 ± 3.2	23.9 ± 4.1	0.907
Smoking(yes/no)	28/32	26/34	0.782
ASA physical status(I/II/III)	13/42/5	15/39/6	0.516
Double-lumen tube size (32/35/37/39-Fr)	8/23/28/1	6/21/31/2	0.819
Type of surgery			0.769
Thoracoscopic lobectomy	46	43	
Thoracoscopic segmental pneumonectomy	14	17	
Side of surgery (left/right)	27/33	25/35	0.643
Duration of anesthesia (min)	154 ± 30	157 ± 33	0.835

BMI: body mass index, ASA physical status: American Society of Anesthesiologists

DLTs passed the trachea more smoothly with lower resistance in the thermal softening group than in the control group (*P* < 0.01). In the thermal softening group, two patients did not require any rotation of the DLT during tube insertion. Other variables related to tracheal intubation were comparable between both groups (Table 2).

**Table 2:** Intubation conditions and hemodynamic responses of the patients to intubation with double-lumen tubes

	Control group (*n* = 60)	Thermal softening group (*n* = 60)	*P*
Mallampati grade (1/2/3)	14/42/4	16/39/5	0.758
Laryngoscopic view (1/2)	53/7	54/8	0.926
Glottis opening score (%)	91(15)	89(18)	0.835
Intubation time (s)	25 ± 8	28 ± 9	0.462
Resistance to advancement of the double-lumen tube through the trachea (None/mild/moderate/severe)	24/18/14/4	48/9/3/0	< 0.001
Mean arterial pressure (mm Hg)			
Before intubation	78 ± 14	80 ± 15	0.529
After intubation	95 ± 19	94 ± 18	0.768
Heart rate (beats min^− 1^)			
Before intubation	68 ± 13	70 ± 12	0.473
After intubation	81 ± 14	82 ± 16	0.784

Values are expressed as number of patients or mean (SD). SD: standard deviation

**Fig. 3:**
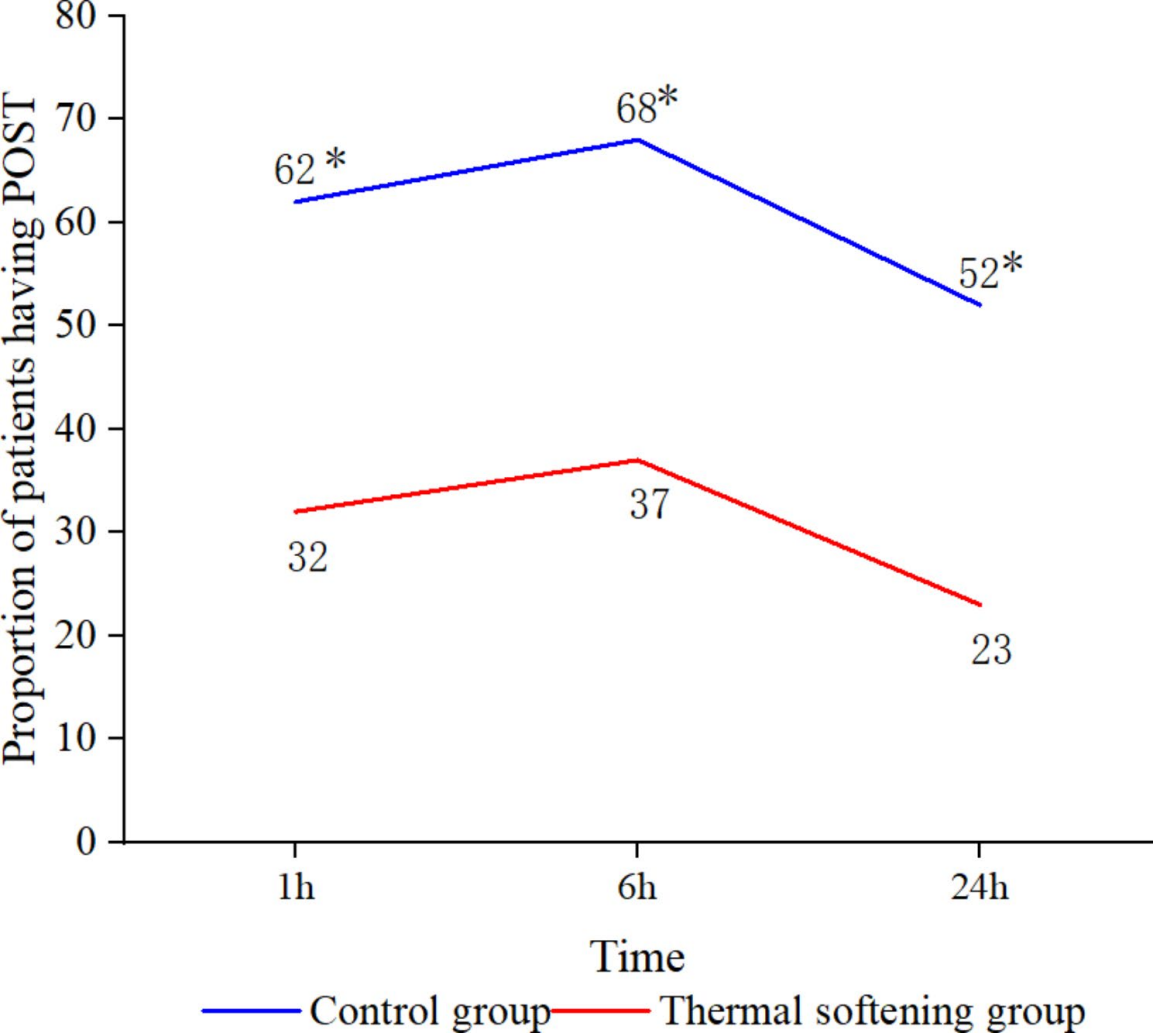
Proportion of patients with POST over time. POST: postoperative sore throat

**Fig. 4:**
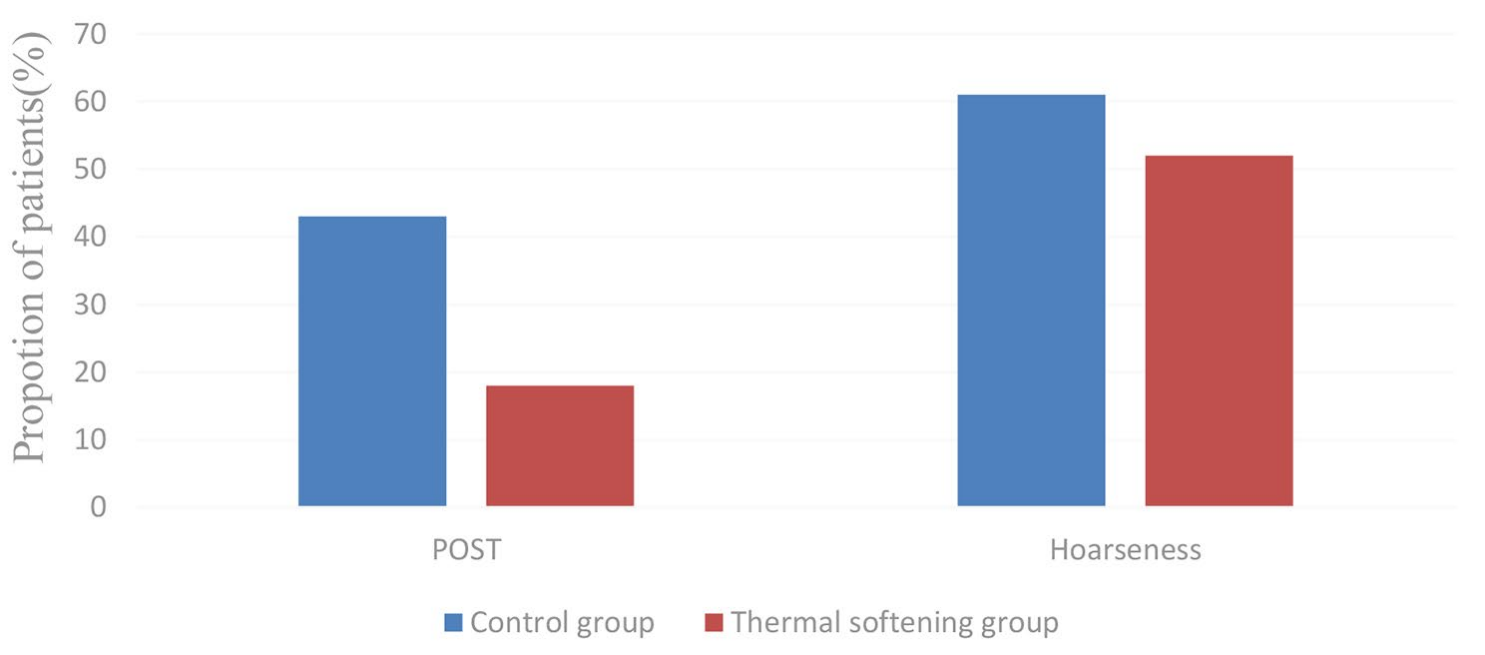
Proportion of patients with severe POST (grade ≥ 2) and hoarseness of voice throughout 24 h after the surgery. POST: postoperative sore throat

The incidence of POST was significantly lower in the thermal softening group than in the control group at 1, 6, and 24 h after the surgery (*P* < 0.05) (Table 3; Fig. 3). The throat pain scores were significantly lower in the thermal softening group than in the control group at 1, 6, and 24 h after the surgery (*P* < 0.05) (Table 3). The incidence of severe POST (grade ≥ 2) throughout 24 h after the surgery was less in the thermal softening group than in the control group (*P* < 0.05) (Fig. 4). Both groups showed a similar incidence of hoarseness at 1, 6, and 24 h after the surgery (Table 3). Moreover, both groups had an equal incidence of oral dryness within 24 h after the surgery (Table 3).

**Table 3:** Incidence and pain score of POST and incidences of postoperative hoarseness and oral dryness ($\bar x \pm s,$  *n* = 60)

	Control group (*n* = 60)	Thermal softening group (*n* = 60)	Risk ratio (95% CI)	*P*
Sore throat incidence				
1 h postoperation	37 (62%)	19 (32%)	0.51 (0.34 to 0.78)	0.001
6 h postoperation	41 (68%)	22 (37%)	0.54 (0.37 to 0.78)	0.001
24 h postoperation	31 (52%)	14 (23%)	0.45 (0.27 to 0.76)	0.001
Overall	46 (76%)	24 (40%)	0.52 (0.37 to 0.73)	< 0.001
Sore throat scores				
1 h postoperation	2 (0, 3)	0 (0, 2)		< 0.001
6 h postoperation	2 (0, 3)	0 (0, 2)		< 0.001
24 h postoperation	2(0, 3)	0 (0, 2)		< 0.001
Hoarseness				
1 h postoperation	39 (65%)	37 (62%)	0.95 (0.72 to 1.25)	0.705
6 h postoperation	32 (53%)	29 (48%)	0.91 (0.64 to 1.29)	0.584
24 h postoperation	21 (35%)	18 (30%)	0.86 (0.51 to 1.44)	0.559
Overall	51(85%)	48 (80%)	0.94 (0.79 to 1.11)	0.471
Oral dryness 24 h	34 (57%)	32 (53%)	0.94 (0.72 to 1.60)	0.714

Values are expressed as median [interquartile range] or number of patients (%). CI: confidence interval

The thermal softening group had a lower total incidence of tracheal injury than the control group(P < 0.05)(Table 4). Moreover, the thermal softening group also had a lower total incidence of vocal cord injury than the control group (*P* < 0.05) (Table 4).

**Table 4:** Incidence of tracheal and vocal cord injuries ($\bar x \pm s,$  *n* = 60)

	Control group (*n* = 60)	Thermal softening group (*n* = 60)	Risk ratio (95% CI)	*P*
Tracheal injury				
Severity				
Mild	35 (58%)	38 (63%)	1.08 (0.81 to 1.44)	0.575
Moderate	16 (27%)	3 (5%)	0.18 (0.05 to 0.61)	0.001
Severe	1 (1.7%)	0 (0.0%)	1.01 (0.98 to 1.05)	0.315
Total incidence	52(86.6%)	41(68.3%)	0.78 (0.64 to 0.96)	0.016
Vocal cord injury				
Edema	14 (23%)	12 (20%)	0.85 (0.43 to 1.69)	0.658
Erythema	16 (26%)	5 (8.3%)	0.31 (0.12 to 0.79)	0.008
Hematoma	3 (5%)	1 (1.7%)	0.33 (0.04 to 3.11)	0.309
Total incidence	33 (55%)	18 (30%)	0.55 (0.35 to 0.86)	0.006

Values are expressed as number of patients (%)

## Discussion

The present study revealed that thermal softening of DLTs before intubation decreased the incidence and severity of POST in patients with prior SARS-CoV-2 infection following thoracoscopic surgery. The airway resistance during insertion and advancement of DLTs also decreased following their thermal softening, and the resulting airway damage also reduced.

The incidence of POST in patients undergoing general anesthesia with DLT is higher than that with SLT; this can reduce postoperative comfort, leading to unpleasantness and irritation in patients after the surgery [21]. SARS-CoV-2 infection causes local mucosal epithelial damage in the pharynx and trachea, resulting in nonspecific inflammation; moreover, in some patients with poor immunity, SARS-CoV-2 infection can persist and lead to chronic inflammation. SARS-CoV-2 mainly invades the airway and can persist in the airway lumen for a long time; this could increase airway sensitivity in patients and induce intubation-related complications. The incidence of POST in the present study was higher than that reported in previous studies; this might be because our patients had prior COVID-19 infection. In the present study, the thermal softening of the DLT prior to intubation decreased airway resistance during the subsequent insertion and advancement of the DLT, decreased airway injuries, and reduced the incidence and severity of POST in patients with prior SARS-CoV-2 infection. The tip of the DLT may be the main cause of resistance and airway injuries during its insertion. Rotation of the tube is often required during DLT insertion. Following its immersion in saline at 50 °C, the DLT becomes softer and more lubricated due to the application of heat; this seems to reduce the damage to the glottis and trachea during the intubation process, thus reducing the incidence of POST. In a previous study, the use of a silicon DLT decreased the incidence of POST compared to the use of a PVC DLT; this might be because of the soft texture of silicone [22].

Because of a larger outer diameter, harder texture, and deeper insertion into the trachea, DLT intubation may cause greater trauma to the airway; however, fewer methods are available to reduce the incidence of POST due to DLTs [4, 22]. Several local anesthetics, systemic analgesics, and corticosteroids have been used to reduce the incidence and severity of POST [23]. Nonpharmacological methods such as 180^0^ rotational advancement [4], jaw thrust maneuver [20], and silicone catheterization [22] may reduce the incidence of POST after DLT intubation. Thermal softening is a simple physical technique that can be performed without any additional support or side effects of drugs.

The vocal cords are the most vulnerable area to sustain injury during tracheal intubation; this is because the glottis is the narrowest part of the upper respiratory tract, and it is closer to the DLT. In our present study, the total incidence of vocal cord injury was 55% in the control group. This incidence was higher than that reported in previous studies [1, 3]; this is possibly because the vocal cords may still be congested and edematous due to the prior SARS-CoV-2 infection. In the present study, the DLT was passed through the glottis and trachea after it was subjected to a heating treatment through immersion in saline at 50 °C; this led to lower resistance during the advancement of the DLT and reduced the incidence of vocal cord and tracheal injuries; these findings indicated that saline immersion and heating of DLT reduced trauma to the throat. To ensure that the anesthesiologist was blinded to the treatment process, only the distal part of the DLT was immersed in saline and heated.

Interestingly, compared to previous studies, the laryngoscope used in the present study was a video laryngoscope rather than a direct laryngoscope; the former provides faster and better exposure of the glottis, with a better laryngoscope field of view and less intubation time, which can reduce the incidence of airway complications [24]. The incidence of POST in the control group was, however, higher than that reported previously. Moreover, the control group showed more severe damage to the vocal cords and trachea than that observed in previous studies. Interestingly, compared to previous studies, the laryngoscope used in the present study was a video laryngoscope rather than a direct laryngoscope; the former provides faster and better exposure of the glottis, with a better laryngoscope field of view and less intubation time, which can reduce the incidence of airway complications [23]. The incidence of POST in the control group was, however, higher than that reported previously. Moreover, the control group showed more severe damage to the vocal cords and trachea than that observed in previous studies [1, 20]. A possible explanation for this finding might be the presence of inflammation and edema in the respiratory mucosa of patients with prior SARS-CoV-2 infection, which probably caused the vocal cords and trachea to become more prone to injury.

The present study has some limitations. First, all tracheal intubations were performed by two anesthesiologists with a rich experience in the intubation of adults with normal airways and were able to intubate quickly. The thermal softening effects may not last long after removal of DLTs from warm saline. Therefore, the findings of this study cannot be generalized to inexperienced physicians and/or patients with difficult airway. Second, although the anesthesiologists were blinded to the study details, they may be able to identify heat-softened DLTs during intubation because of the difference in texture. However, we do not believe that this limitation influenced our results, as we heated only the distal part of the DLT, and the incidence of vocal cord and tracheal injuries is an objective and clear assessment. Third, this study used only one type of polyvinyl chloride DLT for tracheal intubation. Different types of DLTs are prepared from different materials and have varying hardness and tube diameters; this may affect the incidence of POST and airway injury. Further studies are required to determine the optimum approach for inserting different types of DLTs by using a video laryngoscope.

## Conclusions

In conclusion, thermal softening of DLTs before intubation by immersion in a saline solution at 50 °C decreased the incidence and severity of POST in patients with prior SARS-CoV-2 infection following thoracoscopic surgery.

## Data Availability

The datasets used and analysed during this study are available from the corresponding author on reasonable request.

## Abbreviations

BIS: bispectral index
DLTs: double-lumen endobronchial tubes
ETTs: endotracheal tubes
FOB: fiberoptic bronchoscope
PACU: postanesthesia care unit
PCIA: Patient-controlled intravenous analgesia
POST: postoperative sore throat
SARS-CoV-2: severe acute respiratory syndrome coronavirus 2
SLT: single-lumen endobronchial tube
USTC: University of Science and Technology of China
